# Role of the Dopaminergic System in the Acquisition, Expression and Reinstatement of MDMA-Induced Conditioned Place Preference in Adolescent Mice

**DOI:** 10.1371/journal.pone.0043107

**Published:** 2012-08-16

**Authors:** Antonio Vidal-Infer, Concepción Roger-Sánchez, Manuel Daza-Losada, María A. Aguilar, José Miñarro, Marta Rodríguez-Arias

**Affiliations:** Unit of Research on Psychobiology of Drug Dependence, University of Valencia, Valencia, Spain; Dulbecco Telethon Institute at San Raffaele Scientific Institute, Italy

## Abstract

**Background:**

The rewarding effects of 3,4-methylenedioxy-metamphetamine (MDMA) have been demonstrated in conditioned place preference (CPP) procedures, but the involvement of the dopaminergic system in MDMA-induced CPP and reinstatement is poorly understood.

**Methodology/Principal Findings:**

In this study, the effects of the DA D1 antagonist SCH 23390 (0.125 and 0.250 mg/kg), the DA D2 antagonist Haloperidol (0.1 and 0.2 mg/kg), the D2 antagonist Raclopride (0.3 and 0.6 mg/kg) and the dopamine release inhibitor CGS 10746B (3 and 10 mg/kg) on the acquisition, expression and reinstatement of a CPP induced by 10 mg/kg of MDMA were evaluated in adolescent mice. As expected, MDMA significantly increased the time spent in the drug-paired compartment during the post-conditioning (Post-C) test, and a priming dose of 5 mg/kg reinstated the extinguished preference. The higher doses of Haloperidol, Raclopride and CGS 10746B and both doses of SCH 23390 blocked acquisition of the MDMA-induced CPP. However, only Haloperidol blocked expression of the CPP. Reinstatement of the extinguished preference was not affected by any of the drugs studied. Analysis of brain monoamines revealed that the blockade of CPP acquisition was accompanied by an increase in DA concentration in the striatum, with a concomitant decrease in DOPAC and HVA levels. Administration of haloperidol during the Post-C test produced increases in striatal serotonin, DOPAC and HVA concentrations. In mice treated with the higher doses of haloperidol and CGS an increase in SERT concentration in the striatum was detected during acquisition of the CPP, but no changes in DAT were observed.

**Conclusions/Significance:**

These results demonstrate that, in adolescent mice, the dopaminergic system is involved in the acquisition and expression of MDMA-induced CPP, but not in its reinstatement.

## Introduction

The illicit drug MDMA (3,4-methylenedioxy-metamphetamine), known in popular terms as ‘ecstasy’, is a substituted amphetamine whose main effect is a positive mood state encompassing feelings of euphoria, intimacy and closeness to others [1]. While the use of 3,4-methylenedioxymethamphetamine (MDMA) has become more prevalent on a global level, the pattern of consumption has changed considerably. Previously, a subculture of MDMA users was generally restricted to the dance club scene. More recently, use has spread outside of this subculture and many now consume MDMA frequently, with users usually having taken large quantities of pills on at least one occasion and having prolonged their use of the drug for at least 48 h during the previous six-month period [2]. Some users meet the criteria for drug abuse and/or dependence, which become apparent in an association of withdrawal symptoms with abstinence [3].

Addictive drugs increase the levels of synaptic DA in the brain [4], and there is evidence that MDMA induces dopaminergic activity in the mesolimbic reward pathway [5]. MDMA is an indirect monoaminergic agonist [6], [7] that induces the presynaptic release of DA and serotonin (5-HT) [8], [9], [10], [11], [12,], raising extracellular DA and 5-HT in the nucleus accumbens (NAcc) [10], [13], [14] and preferentially increasing DA transmission in the shell rather than to the core of the NAcc [15].

MDMA acts as a reinforcer in both conditioned place preference [16], [17] and self-administration paradigms [18], [19], [20]. Repeated MDMA administration induces sensitization to the behavioral effects of subsequent administrations of the same drug [21], [22] and cross-sensitization to the behavioral effects of cocaine [23] and amphetamines [24]. When administered to mice during adolescence, MDMA increases responsiveness to the rewarding properties of this [25], [26], [27] and other drugs, including morphine [28] and cocaine [29], in adulthood. On the other hand, a priming injection of MDMA can reinstate previously extinguished MDMA self-administration [30] and CPP in rodents [16]. Extinguished MDMA self-administration can also be reinstated by MDMA-conditioned cues [31].

Dopaminergic mechanisms have also been implicated in the positive subjective effects of MDMA. The DA D2 receptor blocker haloperidol attenuated an MDMA-induced positive and mania-like mood but had no influence on other subjective changes provoked by the drug or its cardiovascular effects [32]. Supporting a role for DA in the effects of MDMA, several studies have shown that blockade of dopamine D1 and D2 receptors significantly attenuates MDMA-induced locomotor activity in rats [33], [34], [35]. Moreover, MDMA-induced enhancement of immediate-early genes in the rat striatum is reported to be affected by selective D1 and D2 receptor antagonists [36], [37], although there is evidence that SCH 23390 has only a partial effect [35]. There are few reports of a role for DA in the reinforcing effects of MDMA, and most focus on the self-administration paradigm. For instance, MDMA self-administration is undermined by pre-treatment with the D1 dopaminergic antagonist SCH23390 [38] and the D2 antagonist eticlopride [39]. A recent study has demonstrated the involvement of dopaminergic mechanisms in drug-seeking following extinction of MDMA self-administration [40]. Only one previous study has focused on the role of DA in the acquisition of MDMA-induced CPP in rats, showing that administration of the DA release inhibitor CGS 10746B efficiently blocked an MDMA-induced CPP [41].

The few previous studies to have evaluated the role of DA neurotransmission in the rewarding effects of MDMA have been performed in adult animals, although epidemiological studies show that adolescents are the age group most exposed to this drug [42]. Adolescence is a highly vulnerable developmental period with respect to the consequences of exposure to drugs of abuse [43]. The present study is the first to employ a CPP paradigm to evaluate the role of DA and the main subtypes of DA receptors in the reinforcing properties of MDMA in male adolescent mice. To do this, we administered three DA-specific antagonists: SCH 23390 (preferentially acts at DA D1 receptors), Haloperidol (preferentially acts at DA D2, although it also has a slight effect on D1 receptors), Raclopride (preferentially acts at DA D2 receptors) and the DA release inhibitor CGS 10746B. As the neurotoxic effects of MDMA have been repeatedly demonstrated by a sustained loss in DA, but not in 5-HT, in the mouse striatum [7], [28], [44], alterations in the concentrations of brain monamines were also studied, thus allowing neurochemical and behavioral data to be compared. MDMA binds to the presynaptic serotonin transporter (SERT), inhibiting serotonin reuptake and enhancing the SERT-mediated exchange and release of serotonin. MDMA also enhances the release of dopamine, partially through reversal of dopamine transport, though MDMA itself has a low affinity for dopamine transporters [45]. In light of the mechanism of action of this drug, changes in concentrations of DA (DAT) and serotonin (SERT) transporter were also evaluated.

## Materials and Methods

### Subjects

A total of 486 male mice of the OF1 strain (337 for the CPP experiments and 149 for the neurochemical analyses) were acquired commercially from Charles River (Barcelona, Spain) at 21 days of age. They were housed in groups of four in plastic cages (25×25×14.5 cm) for 5 days before experiments were initiated, under the following conditions: constant temperature (21±2°C), a reversed light schedule (white lights on: 19.30–07.30 h), and food and water available ad libitum, except during behavioral tests. Animals were handled on 2 consecutive days immediately prior to the pre-conditioning (Pre-C) phase in order to reduce their stress levels in response to experimental manipulations. Procedures involving mice and their care were conducted in conformity with national, regional and local laws and regulations, which are in accordance with the European Communities Council Directives (86/609/EEC, 24 November 1986).

### Apparatus

For place conditioning, twelve identical Plexiglas boxes with two equal size compartments (length 30.7 cm, width 31.5 cm, height 34.5 cm) separated by a gray central area (length 13.8 cm, width 31.5 cm, height 34.5 cm) were used. The compartments have different colored walls (black vs white) and distinct floor textures (fine grid in the black compartment and wide grid in the white one). Four infrared light beams in each compartment of the box and six in the central area allowed the position of the animal and its crossings from one compartment to the other to be recorded. The equipment was controlled using three PC computers and MONPRE 2Z software (CIBERTEC, SA, Spain).

### Drugs

Animals were injected i.p. with 10 mg/kg of MDMA (3,4-methylenedioxymetamphetamine hydrochloride, Laboratories Lipomed, Switzerland), 3 and 10 mg/kg of CGS 10746B (Novartis Pharmaceuticals Corporation, Summit, NJ, USA), 0.125 and 0.250 mg/kg of SCH 23390 (Research Biochemical International, Natick, USA), 0.3 and 0.6 mg/kg of Raclopride (RACL) (Astra Laboratory, Sodertalje, Sweden) and 0.1 and 0.2 mg/kg of Haloperidol (HAL) (Laboratorios Sintex Latino S.A, Madrid, Spain). Control groups were injected with physiological saline (NaCl 0.9%), which was also used to dissolve the drugs.

### Procedure of CPP

#### Acquisition

Place conditioning, consisting of three phases, took place during the dark cycle following a procedure that was unbiased in terms of initial spontaneous preference (for more details see [16]. During the first phase, or pre-conditioning (Pre-C), mice were allowed access to both compartments of the apparatus for 15 min (900 s) each day for 3 days. On day 3, the time spent by the animal in each compartment was recorded during a 900 s period. Animals showing strong unconditioned aversion (33% of the session time) or preference (67%) for any compartment were excluded from the rest of the procedure (total number mice excluded discarded from this study = 23 animals). In each group, half the animals received the drug or vehicle in one compartment and the other half in the other compartment. After assigning compartments in this way, an ANOVA showed no significant differences between the time spent in the drug-paired and vehicle-paired compartments during the Pre-C phase. In the second phase (conditioning), animals were conditioned for 30 min a day with MDMA and/or one of the dopaminergic compounds in the drug-paired compartment for 4 days, alternating with saline in the vehicle-paired compartment for another 4 days (a total of 8 days). The dopaminergic drug or saline solution was injected 30 minutes before conditioning, and MDMA was injected immediately prior to conditioning. Access to the central area was cut off by guillotine doors during conditioning. During the third phase, or post-conditioning (Post-C), the guillotine doors separating the two compartments were raised and the time spent by the untreated mice in each compartment was recorded during a 900 s observation period (Post-C tests were performed between 1000 and 1400 hours). The difference in seconds between the time spent in the drug-paired compartment in the Post-C and Pre-C tests is a measure of the degree of conditioning induced by the drug. If this difference is positive, then the drug has induced a preference for the drug-paired compartment, whereas the opposite indicates that an aversion has developed.

To evaluate the effects of the dopaminergic drugs, animals were divided into 18 groups for the conditioning phase: saline (**Sal, n = 10**), CGS 10746B 3 mg/kg (**CGS 3, n = 10**), CGS 10746B 10 mg/kg (**CGS 10, n = 10**), SCH 23390 0.125 mg/kg (**SCH 0.125, n = 9**), SCH 23390 0.250 mg/kg (**SCH 0.250, n = 9**), Raclopride 0.3 mg/kg (**RACL 0.3, n = 9**), Raclopride 0.6 mg/kg (**RACL 0.6, n = 9**), Haloperidol 0.1 mg/kg (**HAL 0.1, n = 9**), Haloperidol 0.2 mg/kg (**HAL 0.2, n = 9**), MDMA 10 mg/kg + saline (**M10, n = 10**), MDMA 10 mg/kg + CGS 10746B 3 mg/kg (**M10+CGS 3 Acq, n = 9**), MDMA 10 mg/kg + CGS 10746B 10 mg/kg (**M10+CGS 10 Acq, n = 8**), MDMA 10 mg/kg + SCH 23390 0.125 mg/kg (**M10+SCH 0.125 Acq, n = 10**), MDMA 10 mg/kg + SCH 23390 0.250 mg/kg (**M10+SCH 0.250 Acq, n = 11**), MDMA 10 mg/kg + Raclopride 0.3 mg/kg (**M10+RACL 0.3 Acq, n = 9**), MDMA 10 mg/kg + Raclopride 0.6 mg/kg (**M10+RACL 0.6 Acq, n = 9**), MDMA 10 mg/kg + Haloperidol 0.1 mg/kg (**M10+HAL 0.1 Acq, n = 8**), and MDMA 10 mg/kg + Haloperidol 0.2 mg/kg (**M10+HAL 0.2 Acq, n = 9**).

#### Expression

To test the effect of the dopaminergic compounds on the expression of the MDMA-induced CPP, all the groups were conditioned with MDMA 10 mg/kg during the acquisition phase and the corresponding dose of one of the dopaminergic drugs was administered 30 minutes before the Post-C test. Animals were divided into 9 groups (n = 10, in al cases): saline (**Sal Exp**), CGS 10746B 3 mg/kg (**CGS 3 Exp**), CGS 10746B 10 mg/kg (**CGS 10 Exp**), SCH 23390 0.125 mg/kg (**SCH 0.125 Exp**), SCH 23390 0.250 mg/kg (**SCH 0.250 Exp**), Raclopride 0.3 mg/kg (**RACL 0.3 Exp**), Raclopride 0.6 mg/kg (**RACL 0.6 Exp**), Haloperidol 0.1 mg/kg (**HAL 0.1 Exp**), and Haloperidol 0.2 mg/kg (**HAL 0.2 Exp**).

#### Extinction and reinstatement

Nine more groups were conditioned with 10 mg/kg of MDMA and underwent a daily extinction session following the Post-C test. Extinction consisted of placing animals in the apparatus (without the guillotine doors separating the compartments) for 900 s until the time spent in the drug-paired compartment by each group was similar to that of the Pre-C test and different from that of the Post-C test. In this way, all the animals in each group were submitted to the same number of extinction sessions, independently of their individual scores. Extinction of CPP was always confirmed in a session 24 hours after the initial extinction session. The effects of a priming dose of MDMA (half of the dose used for conditioning), alone or combined with the dopaminergic compounds, were evaluated 24 hours after extinction was confirmed. The dopaminergic compounds or saline solution were injected 30 minutes before the reinstatement test, which was the same as the Post-C test (free ambulation for 900 s). MDMA was injected 15 minutes before this test began. To evaluate the effects of the dopaminergic compounds on reinstatement, animals were divided into 9 groups according to the drugs administered in the reinstatement test: MDMA 5 mg/kg + saline (**M5−R n = 9**), MDMA 5 mg/kg + CGS 10746B 3 mg/kg (**M5+CGS 3−R, n = 10**), MDMA 5 mg/kg + CGS 10746B 10 mg/kg (**M5+CGS 10−R, n = 10**), MDMA 5 mg/kg + SCH 23390 0.125 mg/kg (**M5+SCH 0.125−R, n = 10**), MDMA 5 mg/kg + SCH 23390 0.250 mg/kg (**M5+SCH 0.250−R, n = 10**), MDMA 5 mg/kg + Raclopride 0.3 mg/kg (**M5+RACL 0.3−R, n = 11**), MDMA 5 mg/kg + Raclopride 0.6 mg/kg (**M5+RACL 0.6−R, n = 10**), MDMA 5 mg/kg + Haloperidol 0.1 mg/kg (**M5+HAL 0.1−R, n = 10**), and MDMA 5 mg/kg + Haloperidol 0.2 mg/kg (**M5+HAL 0.2−R, n = 10**).

### Western Blot Analysis

The concentrations of DAT and SERT were measured in the groups in which we detected blockade of the CPP during the experiments (from n = 13 to n = 5). Ten separate groups of animals received the same corresponding schedules of treatment as in the previous experiment. At the corresponding time of the Post-C test, mice were killed by cervical fracture and their brains quickly removed. The striatum was dissected as described in Heffner et al. [46], and samples were frozen at −80°C until use. Cerebral tissue was homogeneized in 10 volumes of RIPA buffer (150 mM NaCl, 1.0% IGEPAL® CA-630, 0.5% sodium deoxycholate, 0.1% SDS, 50 mM Tris, pH 8.0.) with 10 µl of Dithiothreitol (DTT 1 M), one protease inhibitor tablet (Complete, Mini, EDTA-free Protease Inhibitor Cocktail Tablet, Roche) and one phosphatase inhibitor tablet (PhosStop Phosphatase Inhibitor Cocktail Tablet, Roche) per 10 ml of buffer, and was then incubated under stirring for 30 minutes. The homogenates were centrifuged at 13.000×rpm for 20 min at 4°C, after which the supernatants were collected. The protein concentrations of the lysates were determined using a Bio-RadD protein assay kit (Bio-Rad) and Bovine serum albumin as a standard protein. 10 µg of protein were separated using polyacrylamide gel (Criterion Tris-HCl Glycine 10%) and transferred to a nitrocelulose membrane (Bio-Rad). After blocking the non-specific binding, membranes were probed with polyclonal anti-DAT (1∶6.000; Santa Cruz Biotechnology, Inc) or polyclonal anti-ST (1∶1.500; Santa Cruz Biotechnology, Inc) antibodies in 5% non-fat milk in TBS-T at room temperature overnight. The membranes were then incubated with horseradish peroxidase-conjugated secondary antibodies at room temperature for 2 h, and proteins were detected using SuperSignal West Pico Chemiluminescent Substrate. Western Blotting for actin served as a loading control. Analysis and quantification of the bands was performed using Quantity One software. The DAT/SERT density was divided by the corresponding actin density, and the relative density of DAT and SERT was normalized against that of the control group.

### Analysis of Biogenic Amines

The concentration of brain monoamines was also measured in the groups in which a blockade of the CPP was detected during the experiments (n = 7 in all groups). Ten separate groups of animals received the same schedules of treatment as in the previous experiment, but did not undergo CPP behavioral testing. At the corresponding time of Post-C test, mice were killed by cervical fracture. Within 2 min, their brains were removed and placed on an ice-cold plate. The striatum, hippocampus and cortex were dissected following the procedure described by Heffner et al. [46] and were then frozen on dry ice and stored at −80°C. The tissue was thawed, weighed and then homogenized in 200 µl of perchloric acid (0.1 N) using ultrasounds, and the resulting homogenate was centrifuged at 14.000 rpm for 30 min. The supernatant was divided into aliquots for the analysis of biogenic amines. DA, DOPAC, HVA, 5-HT and 5-HIAA were analyzed in a high performance liquid chromatograph (Agilent 1100 series HPLC). Samples were applied to a column (ZORBAX Eclipse XDB-C8 46×150 mm, 5 µm; Agilent Zorbax High Pressure Cartige Guard-column). A mobile phase consisting of 800 ml of a solution of sodium acetate (0.01 M), 500 ml of a solution of citric acid (0.01 M) ethylenediaminetetraacetic acid disodium salt dehydrate (EDTA, 148 mg) and methanol (255 ml) was passed through the column at a constant flow of 1 ml/min. The HPLC was maintained at a constant temperature (21±1°C). Analytes were oxidized on a glassy carbon electrode maintained at 300 mV (450 mV for HVA detection) against an Ag/AgCl reference electrode (BAS). The complete separation of biogenic amines was achieved in 15 min. Data were collected and analyzed using the Merk-Hitachi software package (Model D-7000). Levels of DA, DOPAC, HVA, 5-HT and 5-HIAA were analyzed in the striatum. In addition, levels of 5-HT and 5-HIAA were analyzed in the cortex and hippocampus.

### Statistical Analysis

To evaluate the acquisition of the CPP, data concerning the time spent in the drug-paired compartment for each of the DA antagonists were analyzed with a mixed ANOVA, with two between subject variables - “MDMA dose”, with two levels (0 and 10 mg/kg), and “Dopaminergic drug dose”, with three levels (No Dose, High Dose, Low Dose) - and a within subject variable - “Days”, with two levels (Pre-C and Post-C). To evaluate the expression of the CPP, data regarding the time spent in the drug-paired compartment for each DA antagonist were analyzed with a mixed ANOVA, with a between subject variable - “Dopaminergic drug dose”, with three levels (No Dose, High Dose, Low Dose) - and a within subject variable - “Days” with two levels (Pre-C and Post-C).

CPP was considered to have been extinguished when the mean time spent by the group in the drug-paired compartment in the extinction session was significantly lower than that in the Post-C test and equal to that in the Pre-C test. These differences were analyzed using Student’s t-tests.

To evaluate the extinction and reinstatement of CPP, the data of the time spent in the drug-paired compartment were analyzed with four mixed ANOVA with a between subjects variable -“Dopaminergic drug dose”, with two levels (High Dose, Low Dose) - and a within subjects variable - “Days”, with four levels (Pre-C, Post-C, Extinction and Reinstatement). Bonferroni tests were used to make post hoc comparisons.

Each monoamine was analyzed using an ANOVA with one between subject variable -“Treatment” - for each of the DA treatment employed: 3 levels for the acquisition study in the case of Haloperidol, Raclopride or CGS 10746B, and 4 levels for SCH 233390. Four levels were employed for the expression study. Bonferroni tests were carried out when appropriate. The differences among the striatum expression levels of DAT and SERT for each dopaminergic drug were analyzed using similar ANOVAS to those employed for brain monoamine concentrations.

**Figure 1:**
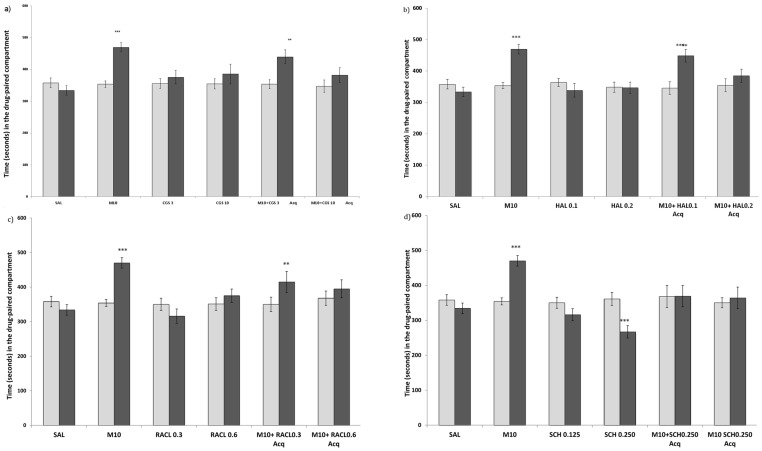
Effects of DA antagonists on the acquisition of MDMA-induced CPP. (1a for the DA release inhibitor CGS 10746B; 1b for the D2 DA antagonist Haloperidol; 1c for the D2 Da antagonist Raclopride; and 1d for the D1 DA antagonist SCH 23390). The bars represent the time in seconds spent in the drug-paired compartment before conditioning sessions during the pre-conditioning test (white bars) and after conditioning sessions during the post-conditioning test (black bars). *** p>0.001; ** p>0.05; significant difference in the time spent in the drug-paired compartment in pre-conditioning vs post-conditioning tests.

## Results

### Acquisition

The ANOVA revealed a significant effect of the interaction MDMA Dose × Dopaminergic drug dose × Days for all the compounds (CGS: F(2,51) = 5,724; p<0,01; HAL: F(2,49) = 3,598; p<0,05; RACL: F(2,50) = 4,254; p<0,05; SCH: F(2,45) = 3,294; p<0.05).

The effects on CPP of DA antagonists, CGS and MDMA 10 mg/kg combined with a DA antagonist or CGS are represented in Figure 1. Post-hoc comparisons indicated that SCH 0.250 mg/kg produced conditioned place aversion (CPA) in animals when administered alone (Fig. 1d). No differences were observed in the groups treated with CGS, Haloperidol or Raclopride alone (Fig. 1a, b and c).

Post-hoc comparisons indicated that animals treated with MDMA 10 mg/kg, MDMA 10 mg/kg + CGS 3 mg/kg, MDMA 10 mg/kg + Haloperidol 0,1 mg/kg or MDMA 10 mg/kg + Raclopride 0,3 mg/kg spent more time in the drug-paired compartment during the Post-C test.

### Expression

**Figure 2:**
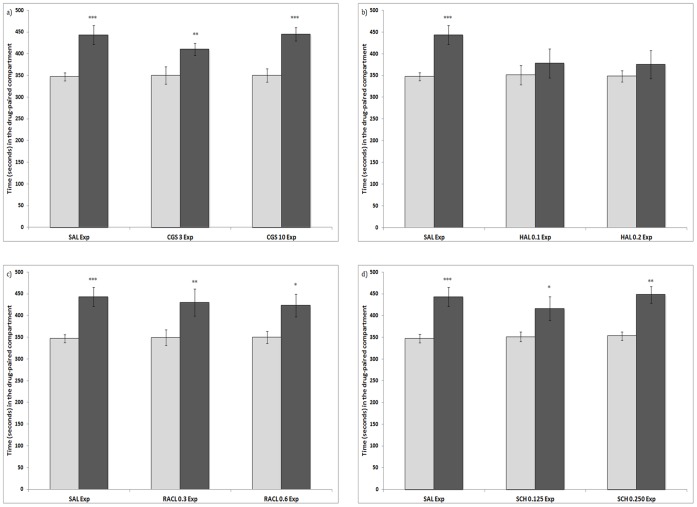
Effects of DA antagonists on the expression of an MDMA-induced CPP. (2a for the DA release inhibitor CGS 10746B; 2b for the D2 DA antagonist Haloperidol; 2c for the D2 Da antagonist Raclopride; and 2d for the D1 DA antagonist SCH 23390). The bars represent the time in seconds spent in the drug-paired compartment before conditioning sessions during the pre-conditioning test (white bars) and after conditioning sessions during the post-conditioning test (black bars). *** p>0.001; ** p>0.01; * p>0.05; significant difference in the time spent in the drug-paired compartment in pre-conditioning vs post-conditioning tests.

The results obtained are shown in Figure 2. The ANOVA revealed that the interaction Dopaminergic drug dose × Days was significant only in the haloperidol group (F(2,24) = 4,312; p<0.05), the development of preference was not observed in the groups treated with this DA antagonist. The variable Days was significant for the rest of the groups, with post-hoc comparisons indicating that the effect of Days was significant, and animals treated with MDMA 10 mg/kg (p<0,001), MDMA 10 mg/kg + CGS 3 mg/kg (p<0,01), MDMA 10 mg/kg + CGS 10 mg/kg, MDMA 10 mg/kg + Raclopride 0,3 mg/kg, MDMA 10 mg/kg + Raclopride 0,6 mg/kg (p<0,05), MDMA 10 mg/kg + SCH 0,125 mg/kg or MDMA 10 mg/kg + SCH 0,250 mg/kg spent more time in the drug-paired compartment during the Post-C test.

### Extinction and Reinstatement

**Figure 3:**
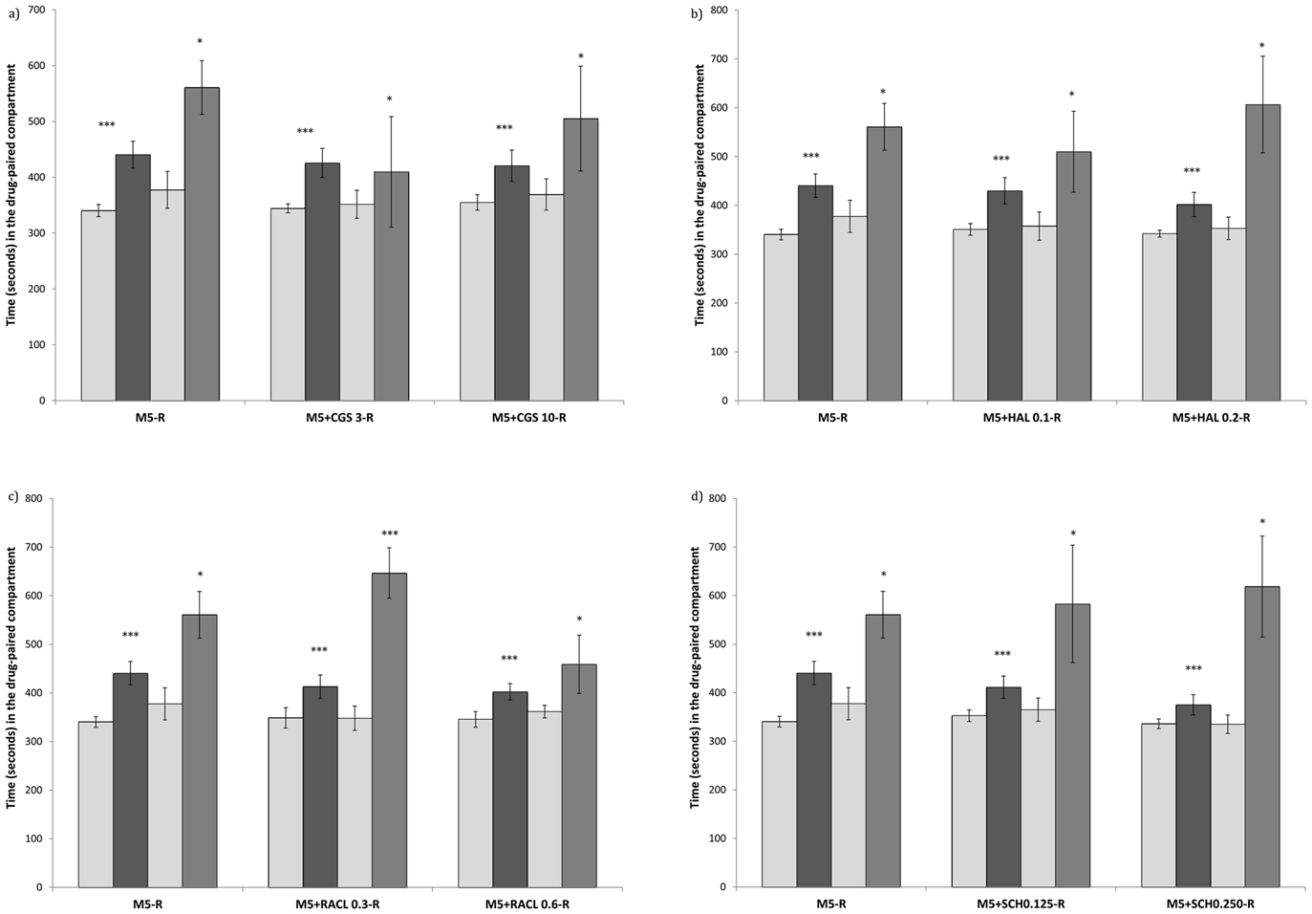
Effects of DA antagonists on the reinstatement of MDMA-induced CPP. (3a for the DA release inhibitor CGS 10746B; 3b for the D2 DA antagonist Haloperidol; 3c for the D2 Da antagonist Raclopride; and 3d for the D1 DA antagonist SCH 23390). The bars represent the time in seconds spent in the drug-paired compartment before conditioning sessions during the pre-conditioning test (white bars), the post-conditioning test (black bars), the extinction test (light gray) and the reinstatement test (dark gray). *** p>0.001; * p<0.05; significant difference in the time spent in the drug-paired compartment in pre-conditioning vs post-conditioning tests.

Extinction of the preference induced by 10 mg/kg of MDMA was achieved after 26±4 sessions. The variable Days was significant for the CGS (F(3,23) = 12,620; p<0,001), haloperidol (F(3,20) = 14,653; p<0,001), raclopride (F(3,21) = 20,618; p<0,001) and SCH )(F(3,21) = 11,247; p<0,001) groups. All the treated mice spent more time in the drug-paired compartment during Post-C and reinstatement tests than in Pre-C and extinction tests. This demonstrates that a priming dose of MDMA (5 mg/kg) induced reinstatement of the extinguished CPP in all the treatment conditions. None of the drugs under study blocked the reinstatement of MDMA-induced CPP. These results are presented in Figure 3.

### Analysis of Biogenic Amines

#### Acquisition

Results are presented in Table 1. For the Haloperidol treatment, the ANOVA for the levels of striatal DA revealed an effect of Treatment (F (2,17) = 15.352; p<0,001), as the animals conditioned with MDMA plus 0.2 mg/kg of Haloperidol presented higher levels of DA than the saline and MDMA groups. For the striatal concentration of DOPAC and HVA, the ANOVA also revealed an effect of treatment (F (2,17) = 11.441; p<0,001), and (F (2,17) = 13.780; p<0,001), since the groups conditioned with saline or MDMA alone presented higher levels of these metabolites than the group treated with haloperidol. DA turnover showed a significant effect for DOPAC/DA (F (2,18) = 20.655; p<0,001) and HVA/DA (F (2,18) = 29.104; p<0,001). DA turnover was lower with Haloperidol treatment than with saline (p<0.001). Equally, biosynthesis of DA, DA+DOPAC (F (2,18) = 3.980; p<0,03) and DO+HVA (F (2,18) = 5.599; p<0,01) was more pronounced in the groups treated with haloperidol than in those treated with saline or M10 (p<0.05).

**Table 1:** Concentration of brain monoamines (acquisition of the CPP).

Striatum	Sal	M10	M10+CGS 10Acq	M10+Hal 0.2Acq	M10+Rac 0.6Acq	M10+SCH 0.125Acq	M10+SCH 0.250Acq
**DA**	12351±378	12616±629	15413±640**	16482±1043**	14565±565 *	16810±717**	14859±574 **
**DOPAC**	2070±154	2049±198	1342±84*	966±108**	687±71**	1299±151**	832±65**
**HVA**	1475±85	1137±48	1199±96	830±100**	885±53**	1206±102	812±55**
**DOPAC/DA**	0,17±0.01	0,16±0.01	0,09±0.002***	0,06±0.004***	0,05±0.004***	0,08±0.01***	0,05±0.003***
**HVA/DA**	0,12±0.006	0,09±0.004	0,08±0.01***	0,04±0.01***	0,06±0.002***	0,07±0.005***	0,05±0.002***
**DA + DOPAC**	14422±410	14665±709	16756±748*	17449±1172*	15252±620	18110±752***	15691±633
**DA + HVA**	13827±433	13753±672	16613±728*	17312±1161*	15450±631	18016±812***	15672±634
**5-HT**	759±31	758±72	789±37	846±42	916±64	1020±70	976±81
**5-HIAA**	444±37	311±32*	242±32**	272±25**	196±18**	247±30**	236±25**
**5-HIAA/5-HT**	0,56±0.04	0,4±0.06	0,3±0.04**	0,3±0.02**	0,2±0.01***	0,2±0.02***	0,2±0.03***
**5-HT +5-HIAA**	1203±58	1070±75	1031±48	1118±59	1112±79	1267±90	1212±81
*Frontal cortex*							
**5-HT**	771±40	797±42	625±42*	705±63	701±41	640±83	750±44
**5-HIAA**	140±9	149±12	144±14	122±11	93±6 **	111±17	112±7
**5-HIAA/5-HT**	0,18±0.008	0,19±0.01	0,23±0.01*	0,18±0.02	0,13±0.006**	0,18±0.01	0,15±0.009
**5-HT +5-HIAA**	911±46	947±51	769±51	828±69	795±44	752±96	862±46
*Hippocampus*							
**5-HT**	397±28	420±22	398±20	542±54 *	447±38	467±22	537±48 *
**5-HIAA**	357±24	288±21	201±13**	268±28	239±28*	248±17*	171±12**
**5-HIAA/5-HT**	0,91±0.07	0,69±0.03*	0,51±0.04***	0,5±0.04***	0,53±0.04***	0,45±0.07***	0,33±0.03***
**5-HT +5-HIAA**	754±43	709±38	599±25	811±75	686±59	715±67	709±49

Mice were treated during the conditioning phase of the CPP with saline (Sal), MDMA 10 mg/kg + saline (M10), MDMA 10 mg/kg + CGS 10746B 10 mg/kg (M10+CGS 10 Acq), MDMA 10 mg/kg + Haloperidol 0.2 mg/kg (M10+HAL 0.2 Acq), MDMA 10 mg/kg + Raclopride 0.6 mg/kg (M10+RACL 0.6 Acq), MDMA 10 mg/kg + SCH 23390 0.125 mg/kg (M10+SCH 0.125 Acq), and MDMA 10 mg/kg + SCH 23390 0.250 mg/kg (M10+SCH 0.250 Acq). Data are presented as means with ±S.E.M. Differences with respect to the saline group.

* p<0.01,

** p<0.001.

The striatal concentration of 5-HIAA (F (2,17) = 6.767; p<0,007) showed lower concentrations in both groups treated with MDMA than in that treated with saline. Serotonin turnover in the striatum (F (2,18) = 7.262; p<0,005) was lower in haloperidol-treated mice than in controls (p<0.01). Levels of serotonin in the hippocampus (F (2,17) = 4.453; p<0,02) were higher in the group treated with haloperidol that in the control group. In line with this, serotonin turnover in the hippocampus (F (2,18) = 13.843; p<0,001) was lower in MDMA- and haloperidol-treated mice than in controls (p<0.05 for M10 and p<0.001 for M10+Hal 0.2Acq).

For SCH 23390, the ANOVA for levels of striatal DA revealed an effect of Treatment (F (3,23) = 9.438; p<0,001), as the animals conditioned with MDMA plus any of the doses of SCH 23390 presented higher levels of DA than those receiving saline. The ANOVA also revealed an effect of treatment for the striatal concentration of DOPAC and HVA (F (3,23) = 14.126; p<0,001), and (F (3,23) = 11.285; p<0,001), since the groups treated with this DA D1 antagonist presented lower levels of DOPAC than those treated with saline or MDMA. The concentration of HVA was lower in animals treated with the higher doses of SCH 23390 than in saline controls. DA turnover showed a significant effect for DOPAC/DA (F (3,24) = 19.533; p<0,001) and HVA/DA (F (3,24) = 27.890; p<0,001). Both doses of SCH 23390 resulted in lower DA turnover than in the saline-treated group (p<0.001). Equally, biosynthesis of DA, DA+DOPAC (F (3,24) = 6.641; p<0,001) and DA+HVA (F (3,24) = 9.088; p<0,001) was more pronounced in the group treated with the lowest dose of SCH 23390 than in the saline or M10 groups (p<0.001).

The striatal concentration of 5-HIAA (F (3,23) = 8.278; p<0,001) was lower in both the groups treated with SCH22390 than in saline-treated mice. Serotonin turnover in the striatum (F (3,24) = 12.575; p<0,001) was lower in both the groups treated with SCH 23390 than in controls or MDMA-treated mice (p<0.001 for saline and p<0.03 for M10).

Serotonin in the hippocampus (F (3,23) = 3.561; p<0,03), was higher in the group treated with the highest dose of SCH 23390 than in controls However, concentration of 5-HIAA in the same structure (F (3,23) = 15.721; p<0,001) was lower in mice treated with any dose of SCH 23390 than in controls. Consequently, serotonin turnover in the hippocampus (F (3,24) = 18.482; p<0,001) was lower in both the groups treated with SCH 23390 than in controls (p<0.001).

In the case of Raclopride, the ANOVA for the levels of striatal DA revealed an effect of Treatment (F (2,18) = 4.564; p<0,02), as the animals conditioned with MDMA plus Raclopride presented higher levels of DA than those receiving saline. The ANOVA also revealed an effect of treatment for the striatal concentration of DOPAC and HVA (F (2,18) = 24.777; p<0,001), and (F (2,18) = 19.217; p<0,001), since the group treated with this DA D2 antagonist presented lower levels of DA metabolites than those treated with saline or MDMA. DA turnover showed a significant effect for DOPAC/DA (F (2,18) = 24.822; p<0,001) and HVA/DA (F (2,18) = 34.229; p<0,001). DA turnover was lower in the Raclopride-treated group than in those treated with saline or MDMA alone (p<0.001).

The concentration of 5-HIAA in the striatum (F (2,18) = 16.344; p<0,001), frontal cortex (F (2,18) = 10.069; p<0,001) and hippocampus (F (2,18) = 5.636; p<0,01), was lower than in saline-treated mice. Serotonin turnover in the striatum (F (2,18) = 15.941; p<0,001), cortex (F (2,18) = 11.333; p<0,001) and hippocampus (F (2,18) = 11.694; p<0,001) was lower in the group treated with raclopride than in that treated with saline (p<0.01 for cortex and p<0.001 for striatum and hippocampus).

For CGS 10746B, the ANOVA for the levels of striatal DA revealed an effect of Treatment (F (2,18) = 8.132; p<0,003), as the animals treated with this drug presented higher levels of DA than saline controls. The striatal concentration of DOPAC in the groups treated with CGS 10746B (F (2,18) = 6.571; p<0,007) was lower than in the groups treated with saline or MDMA. DA turnover showed a significant effect for DOPAC/DA (F (2,18) = 11.487; p<0,001) and HVA/DA (F (2,18) = 14.095; p<0,001). Administration of CGS 10746B resulted in lower DA turnover than that of saline (p<0.001). Equally, biosynthesis of DA, DA+DOPAC (F (2,18) = 3.847; p<0,04) and DA+HVA (F (2,18) = 6.538; p<0,007) was more marked in the group treated with CGS 10746B than in saline-treated animals (p<0.05 for DA+DOPAC and p<0.01 for DA+HVA).

Concentration of serotonin in the frontal cortex (F (2,18) = 4.848; p<0,02) was lower in the groups treated with CGS 10746B than in those treated with MDMA alone. The concentration of 5-HIAA in the striatum (F (2,18) = 8.901; p<0,002) and hippocampus (F (2,18) = 14.524; p<0,0011) was lower than in saline-treated mice. Serotonin turnover in the striatum (F (2,18) = 6.619; p<0,04) and hippocampus (F (2,18) = 13.886; p<0,04) was lower in the group treated with the DA release inhibitor (p<0.001). However, serotonin turnover in the cortex (F (2,18) = 4.369; p<0,02) increased after CGS 10746B treatment (p<0.05).

#### Expression

Results are presented in Table 2. DA concentration in the striatum did not show any significant effect, but the ANOVA for the levels of striatal DOPAC (F (3,28) = 94.212; p<0,001) and HVA (F (3,28) = 40.553; p<0,001) revealed that Treatment did have an effect. Animals injected with any one of the doses of Haloperidol presented higher levels of DOPAC and HVA than those in the other groups (p>0.001 in all cases). DA turnover showed a significant effect for the ratio DOPAC/DA (F (3,28) = 52.677; p<0,001) and HVA/DA (F (3,28) = 54.811; p<0,001). Both doses of haloperidol led to a higher DA turnover than treatment with saline or MDMA alone (p<0.001 in both cases). Equally, biosynthesis of DA, DA+DOPAC (F (3,28) = 17.124; p<0,001) and DA+HVA (F (3,28) = 8.829; p<0,001) was higher in both the groups treated with haloperidol than in saline- or M10-treated animals (p<0.001).

**Table 2:** Concentration of brain monoamines (expression of the CPP).

Striatum	Sal	M10	M10+Hal 0.1Exp	M10+Hal 0.2Exp
**DA**	12351±378	12616±629	14674±662	14720±795
**DOPAC**	2070±154	2049±198	4094±228**	5923±145**
**HVA**	1475±85	1137±48	2480±169**	3513±265**
**DOPAC/DA**	0,17±0.01	0,16±0.01	0,4±0.01***	0,28±0.01***
**HVA/DA**	0,12±0.061	0,09±0.004	0,24±0.01***	0,17±0.01***
**DA + DOPAC**	14422±410	14665±709	18768±802***	20643±899***
**DA + HVA**	13827±433	13753±672	17154±785***	18234±1018***
**5-HT**	759±31	758±72	1131±60**	996±82*
**5-HIAA**	444±37	311±32*	362±38	328±27
**5-HIAA/5-HT**	0,58±0.04	0,44±0.06	0,35±0.04**	0,32±0.02**
**5-HT +5-HIAA**	1203±58	1070±75	1494±90	1325±82
*Frontal cortex*				
**5-HT**	771±40	797±42	659±60	782±14
**5-HIAA**	140±9	149±12	144±17	148±10
**5-HIAA/5-HT**	0,18±0.01	0,19±0.01	0,19±0.01	0,22±0.02
**5-HT +5-HIAA**	911±46	947±51	804±70	930±20
*Hippocampus*				
**5-HT**	397±28	420±22	374±52	428±30
**5-HIAA**	357±24	288±21	171±46**	219±8*
**5-HIAA/5-HT**	0,91±0.01	0,69±0.03	0,52±0.02*	0,78±0.4
**5-HT +5-HIAA**	754±42	709±38	545±38	648±36

Mice were treated on the test day of the CPP with saline (Sal), MDMA 10 mg/kg + saline (M10), MDMA 10 mg/kg + Haloperidol 0.1 mg/kg (M10+HAL 0.1 Acq), and MDMA 10 mg/kg + Haloperidol 0.2 mg/kg (M10+HAL 0.2 Acq). Data are presented as means with ±S.E.M. Differences with respect to the saline group.

* p<0.01,

** p<0.001.

Concentration of serotonin in the striatum also revealed an effect of Treatment (F (3,28) = 8.007; p<0,001). In the animals treated with haloperidol higher levels of serotonin were observed with respect to those in the Sal and M10 groups (p<0.01 for the higher dose and p<0.001 for the lower dose). No differences were observed between concentrations of 5-HIAA in the striatum. Consequently, serotonin turnover (F (3,28) = 6.188; p<0,001) was lower in both the groups treated with haloperidol than in the saline-treated group (p<0.01).

No differences were observed in frontal cortex levels of serotonin or 5-HIAA.

Serotonin concentration in the hippocampus was not found to have a significant effect, but the ANOVA for the levels of hippocampus 5-HIAA revealed an effect of treatment (F (3,28) = 7.884; p<0,001), with lower concentrations being detected in mice treated with haloperidol than in those treated with saline (p<0.001 for the lower and p<0.01 for the higher dose of haloperidol). For this reason, serotonin turnover (F (3,28) = 5.105; p<0,001) was lower in the group treated with the lowest dose of haloperidol than in the saline- and MDMA-treated groups (p<0.01 for Saline and p<0.05 for M10).

### Western Blot

The ANOVA for Haloperidol (F (2,23) = 3.894; p<0,03) when administered during the acquisition of the CPP revealed an effect of treatment on SERT concentration, as higher levels were observed in the M10+Hal 0.2 Acq group than in saline-treated animals.

**Figure 4:**
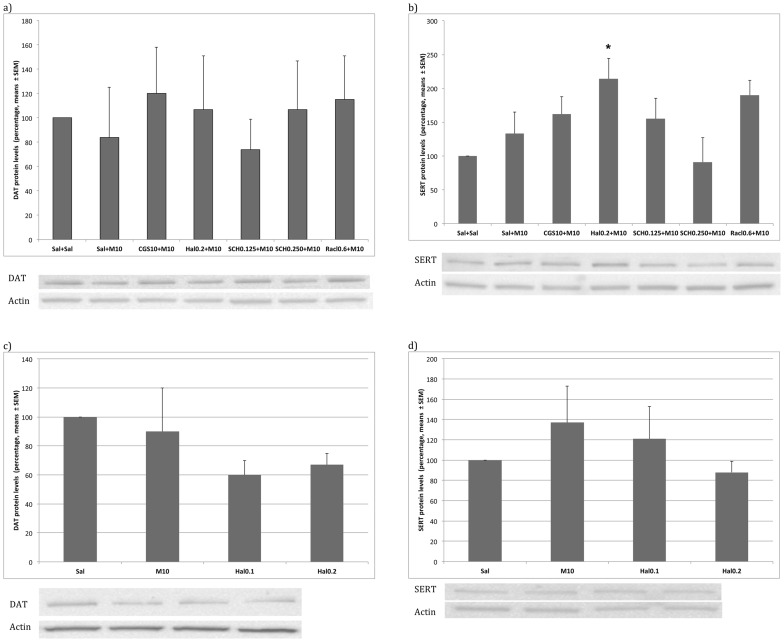
Concentration of the Actin DA transporter DAT and the serotonin transporter SERT. Mice were treated during the acquisition or expression phase of the MDMA-induced CPP. In the acquisition phase (4a and 4b), mice were treated during the conditioning phase of the CPP with saline (Sal, n = 12), MDMA 10 mg/kg + saline (M10, n = 13), MDMA 10 mg/kg + Haloperidol 0.2 mg/kg (M10+HAL 0.2 Acq, n = 6), MDMA 10 mg/kg + SCH 23390 0.125 mg/kg (M10+SCH 0.125 Acq, n = 5), MDMA 10 mg/kg + SCH 23390 0.250 mg/kg (M10+SCH 0.250 Acq, n = 6), MDMA 10 mg/kg + Raclopride 0.6 mg/kg (M10+RACL 0.6 Acq, n = 7), and MDMA 10 mg/kg + CGS 10746B 10 mg/kg (M10+CGS 10 Acq, n = 7). In the expression phase (4c and 4d) mice were treated on the test day of the CPP with (Sal, n = 12), MDMA 10 mg/kg + saline (M10, n = 13), MDMA 10 mg/kg + Haloperidol 0.1 mg/kg (M10+HAL 0.1 Exp, n = 8), and MDMA 10 mg/kg + Haloperidol 0.2 mg/kg (M10+HAL 0.2 Exp, n = 8). Data are presented as means with ±S.E.M. The images which can be seen belong to the same gel. Differences with respect to the saline group *p<0.05, ** p<0.01.

DAT and SERT densities did not differ in the expression of the CPP. These data are presented in Figure 4.

Reprobing of blots for actin confirmed equal protein loading across groups.

## Discussion

Our results confirm that DA transmission plays a critical role in the acquisition and expression of MDMA-induced CPP in adolescent mice. Blockade of D1, D2 or both DA receptors at doses that did not induce motivational effects during the conditioning phase efficiently impeded the development of an MDMA-induced CPP. Similar results were obtained with the DA release inhibitor CGS 10746B. Moreover, expression of the CPP was blocked only by haloperidol. No role was found for DA neurotransmission in the reinstatement of an MDMA-induced CPP, since none of the drugs employed were capable of blocking drug priming-induced reinstatement.

It is assumed that the place conditioning paradigm reflects the secondary motivational properties of drugs and their potential for abuse [47], [48]. In accordance with this hypothesis, some DA antagonists can produce CPA, while the administration of 10 mg/kg of MDMA has been reported to cause CPP in mice [16], [27]. In the present study, administration of different DA antagonists alone did not produce motivational effects, except in the case of the D1 antagonist SCH 23390. At the highest dose employed (0.25 mg/kg), blockade of DA D1 receptors induced place aversion, a result that is in line with the findings of both classic and more recent reports [49], [50], [51].

### Role of DA in the Acquisition of MDMA-induced CPP

The dopaminergic system is known to play a critical role in the acquisition of the CPP induced by different drugs. Morphine-induced CPP is blocked when SCH 23390, haloperidol or raclopride is administered during the conditioning phase [52]. A role for the D1 DA receptor has also been identified in cocaine-induced CPP, as SCH 23390 efficiently blocks the development of preference [53]. In line with this, cocaine self-administration is absent in D1 receptor knockout mice [54], [55], [56]. Although there are no previous reports of a role of DA receptors in MDMA-induced CPP, past self-administration studies have found that both D1-like and D2-like receptors contribute to the maintenance of MDMA self-administration [38], [39]. Our results confirm the importance of the role of the DA D1 receptor in the acquisition of MDMA-induced CPP, since we found that the specific antagonist SCH 23390 effectively blocked said acquisition at doses that did not induce motivational effects. Similarly, administration of the highest dose of the DA D2 antagonist Raclopride blocked the MDMA-induced CPP. These results endorse the hypothesis which holds that DA D2 receptors are as important as DA D1 receptors in reward. Equally, the development of CPP was blocked by the highest dose of haloperidol administered during the conditioning process. In this way, the present findings demonstrate that D1 and D2 DA receptors are necessary for the acquisition of MDMA-induced CPP, as they are for other drugs of abuse.

CGS 10746B is known to attenuate the release of dopamine without binding to synaptic dopamine receptor sites [57]. Accordingly, in a previous study we observed that this drug had no rewarding or aversive properties at the doses tested, as they failed to affect place conditioning [52]. In addition, earlier reports have implicated DA release in the CPP induced by heroin [49], cocaine and MDMA [41]. The present results support these past findings and bolster the idea that the rewarding effects of MDMA in the place conditioning paradigm are mediated essentially by the DA system also in adolescent animals.

Several processes are crucial to the acquisition of a CPP. In basic terms, the stimulus must be rewarding and the animal must associate these rewarding effects with environmental cues, which implies an associative learning. Disruption of one of these processes will be manifested as a reduced preference for the drug-paired compartment. The fact that DA antagonists and inhibition of DA release block the acquisition of an MDMA-induced CPP means that this neurotransmitter is critical to one or both of the aforementioned processes. Similarly to that observed with other addictive drugs, microdialysis studies have shown a preferential increase in synaptic DA in the nucleus accumbens shell following exposure to MDMA [15]. A single dose of 10 mg/kg of MDMA provoked increases in synaptic DA that were comparable to those produced by the same dose of other stimulants used in drug discrimination studies [58], [59], [60]. Thus, disruption of DA neurotransmission can impede animals from experiencing the reinforcing effect of MDMA. On the other hand, CPP can be viewed as an incentive learning task. Accordingly, during pairing sessions, the stimuli on the side associated with reward acquire an increased ability to elicit approach and other responses. As a result of this learning, animals spend more time there during the test [61]. Incentive learning may be critically dependent on the action of DA on D1-receptors. In addition, the D1 antagonist SCH-23390 blocks not only acquisition of the conditioned place-preference induced by various drugs, but also the acquisition of conditioned taste aversion by disrupting the formation of a short-term memory trace of the gustatory conditioned stimulus. However, no effect has been observed when a DA D2 receptor is blocked with raclopride [62].

As we have previously reported, the CPP induced by 10 mg/kg of MDMA did not induce a decrease in DA or any alteration in serotonin or its metabolites [16]. Monoamines levels were determined 24 h after the last drug administration, a fact that can be responsible for, among other factors, the lack of an increase in DA or serotonin in the groups treated with MDMA. It should be taken into consideration that the procedure we have employed allows the detection of monoamine levels in the brain structure under study, but does not allow their origin (intra or extra neuronal) to be determined. Blockade of MDMA-induced CPP by any of the dopaminergic drugs employed was accompanied by an increase in DA concentration in the striatum and a concomitant decrease in DOPAC levels, which decrease DA turnover. An increase in DA biosynthesis was also observed after treatment with CGS 10746B, haloperidol and SCH 23390. A similar reduction was also observed in serotonin turnover in the striatum and hipoccampus due to marked decreases of its metabolite 5-HIIA or increases in its concentration in the hippocampus. These results are not surprising in light of the previous observation that acute administration of neuroleptics increases dopaminergic neuronal firing and augments synaptic release of dopamine [63], [64], [65] via blockade of DA receptors or autoreceptors [66], [67]. The present results suggest that all DA antagonists and the DA release inhibitor CGS 10746 alter the DA system profoundly at doses that inhibit the development of an MDMA-induced CPP, thereby producing an increase in the total amount of striatal DA while not affecting serotonin levels in any obvious way.

No changes were observed in the concentration of DA or serotonin transporters, although all the drugs administered tended to increase SERT concentration, with Haloperidol producing the strongest rise.

### Role of DA in the Expression of MDMA-induced CPP

The CPP procedure is useful for distinguishing the effects of drugs on acquisition and those on expression of learning. Acquisition takes place during the pairing sessions, and expression is evaluated on the day following conditioning. Experimental drugs can be administered during the acquisition phase, or, alternatively, experimental compounds can be given with the rewarding drug alone on the test day after conditioning. If CPP is not observed, the experimental compound is considered to have blocked the expression of CPP. The present study shows that DA is only involved in the expression process when both DA receptors, D1 and D2, are antagonized simultaneously. We observed that only haloperidol (0,1 and 0,2 mg/kg) blocked MDMA-induced CPP expression. Moreover, our results are similar to those of Adams and co-workers [68], who reported that haloperidol significantly blocked the expression of cocaine-CPP in rats, while raclopride and SCH 23390 did not. Other studies have demonstrated that a simultaneous antagonism of D1 and D2/D3 receptors is required in order to block expression of a cocaine-CPP [69], [70], which implicates conditioned DA release in expression mechanisms.

No effect was observed in the groups treated with CGS 10746B, for which there are several possible explanations. Firstly, it is possible that DA release does not occur during the expression of the CPP, which would be in line with a recent report by Weitemier and Murphy [71]. They observed that extracellular DA was not enhanced with respect to control levels during the expression phase of morphine-induced CPP. Secondly, at the doses administered in the present study, CGS 10746B does not produce motor impairment [49], but haloperidol significantly undermines motor activity [72]. Accordingly, the number of crossings between the two compartments during the expression test was significantly lower among mice treated with haloperidol than in saline- or CGS 10746B-treated animals. Therefore, the approach behavior of mice to reward cues could be affected following haloperidol administration, resulting in blockade of the MDMA-induced expression of the CPP. In this context, we should take into consideration that an impairment of motor performance could be responsible for the blockade observed.

Animals treated with haloperidol on the day of the test presented similar levels of DA to those of controls and animals treated with 10 mg/kg of MDMA, although their levels of DOPAC and HVA were higher, and this led to an increase in DA turnover. In addition, these mice exhibited higher levels of serotonin in the striatum without exhibiting changes in their metabolite concentration. In this way, the animals that did not show a preference for the compartment associated with MDMA after being treated with haloperidol during the test presented an increased DA turnover and a decreased serotonin turnover in the striatum. The differences with the results obtained after administration of haloperidol during the acquisition phase could be due to the fact that four haloperidol doses were administered in the acquisition study and that monoamines were measured 24 hours after the last injection (i.e., on the day of the test). A second possible explanation is that, in the expression study, measurement of monoamines took place 30 minutes after a single injection of the neuroleptic. Moreover, no changes were observed in the concentration of the transporters (DAT and SERT).

### Role of DA in the Reinstatement of MDMA-induced CPP

Memories of the learned association between cues and the rewarding properties of abused drugs are difficult to extinguish and contribute significantly to the high propensity to relapse [73]. The results of the present study demonstrate that the dopaminergic system is not involved in the reinstatement of MDMA-induced CPP in adolescent mice. In accordance with previous results [16], [26], [27], [74], [75], [76], after extinction of a MDMA-induced CPP, a priming dose of 5 mg/kg of MDMA produced reinstatement of the preference in adolescent mice, even when combined with any of the DA antagonists or the DA release inhibitor.

When compared with other drugs of abuse, there is a relative paucity of studies that have assessed drug-seeking following MDMA self-administration. In a recent report, Schenk and coworkers [40] observed that pretreatment with either SCH 23390 or the D2-like receptor antagonist eticlopride undermined the response produced by a priming injection of MDMA. The authors suggested that there had been a shift in the 5-HT/dopamine effects of MDMA as a result of self-administration, since this procedure induces a decrease in brain tissue levels of 5-HT. As a consequence, synaptic dopamine could have been enhanced, which, in turn, would lead to drug-taking and drug-seeking being controlled by dopaminergic mechanisms. If so, pharmacological manipulations of dopaminergic systems would be expected to affect drug-seeking. The fact that MDMA did not affect DA or serotoninergic levels in our study could explain the discrepancy between our results and those of other authors.

Although most of the results obtained using the CPP model of reinstatement confirm those of self-administration studies, some inconsistencies have emerged. These may be due to either a difference in methodology – for example, the animals (species, strain, age and sex) and drug doses employed – or the different response requirements used to assess reinstatement (lever pressing behavior versus stay in a chamber previously paired with the drug). Moreover, it should be taken into account that CPP and self-administration paradigms evaluate different aspects of reward and, thus, different characteristics of relapse and addictive behavior [77]. Similarly to that observed in the present study, self-administration experiments have demonstrated that the manipulation of DA neurotransmission blocks heroin-induced reinstatement of self-administration [74], [75], [76], while Ribeiro Do Couto and cols. [78] showed that SCH 23390, Raclopride, Haloperidol and CGS 10746B fail to block the reinstatement of CPP induced by morphine primes.

In conclusion, the present study demonstrates that the dopaminergic system is involved in MDMA-induced CPP acquisition and expression, but not in reinstatement, in adolescent mice. The combined effects of the dopaminergic, serotoninergic, glutamatergic and cannabinoid systems should be evaluated in future studies, as knowledge of the neurobiological basis of the reinforcing effects of MDMA will undoubtedly be of help in designing adequate therapies for ecstasy users that develop dependence or are concerned about their use of MDMA.
